# P300/CBP inhibition with inobrodib in combination with gilteritinib and venetoclax targets leukemia stem cells in epigenetic mutant AML

**DOI:** 10.1126/sciadv.aec9305

**Published:** 2026-05-15

**Authors:** Melanie L. Goetz, Jennifer S. Romer-Seibert, Amanda M. Versace, Scott Kogan, Chetan Jeurkar, Robert L. Bowman, Nigel Brooks, Kris Frese, Sara E. Meyer

**Affiliations:** ^1^ Department of Pharmacology, Physiology, and Cancer Biology, Sidney Kimmel Comprehensive Cancer Center, Thomas Jefferson University, Philadelphia, PA, USA.; ^2^ Department of Laboratory Medicine and Helen Diller Family Comprehensive Cancer Center, University of California, San Francisco, San Francisco, CA, USA.; ^3^ Division of Hematologic Malignancies, Department of Medical Oncology, Sidney Kimmel Comprehensive Cancer Center at Thomas Jefferson University, Philadelphia, PA, USA.; ^4^ Department of Cancer Biology, University of Pennsylvania, Philadelphia, PA, USA.; ^5^ CellCentric Ltd., Cambridge, UK.; ^6^ Department of Medical Oncology, Sidney Kimmel Comprehensive Cancer Center, Thomas Jefferson University, Philadelphia, PA, USA.

## Abstract

Acute myeloid leukemia (AML) is a fatal blood cancer with cytotoxic chemotherapy offering at best 25% 5-year survival. While targeted BCL2 and FLT3 inhibitors venetoclax and gilteritinib are used upfront in the treatment of a subset of adult patients with AML and help to extend the survival of some patients, a curative treatment combination with minimal side effects has yet to be discovered. We find that use of the dual histone acetyltransferase p300/CBP bromodomain inhibitor CCS1477 (inobrodib), together with venetoclax and gilteritinib, virtually eliminates leukemia stem cells in an aggressive preclinical model of *DNMT3A/FLT3*-mutant AML by impairing pro-oncogenic survival and proliferation factors to effectively block leukemogenesis. This work identifies potential clinical utility of a targeted, triplet combination therapy for treatment of AML.

## Teaser

Combination therapy wipes out myeloid leukemia stem cells, paving the way toward clinical translation.

## INTRODUCTION

Epigenetic deregulation is recognized as a hallmark of hematologic malignancies. The ability to selectively reverse oncogenic gene expression programs underlies the rational targeting of epigenetic regulators for treatment of cancers, including acute myeloid leukemia (AML). Among the family of histone acetyltransferases, p300 and CBP are the two highly homologous transcriptional coactivators known to play important roles in leukemogenesis by regulating cell proliferation, apoptosis, and differentiation (*1*–*3*). The small-molecule CCS1477 (inobrodib) is highly specific to the bromodomains of p300 and CBP, which tether them to DNA (*4*, *5*). CCS1477 showed promising activity in preclinical models of prostate cancer, multiple myeloma, and AML (*4*, *5*). Moreover, CCS1477 is now in early-phase clinical trials for treatment of relapsed or refractory hematologic malignancies (NCT04068597) (*5*). In AML, CCS1477 promotes myeloid differentiation of primary AML samples in vitro and in select patients with AML from the phase 1 study receiving CCS1477 as monotherapy (*5*). Thus, CCS1477 is well tolerated in patients with AML and may have therapeutic utility to induce differentiation (*5*). Moving forward, it is imperative to identify potential biomarkers of patients who might benefit from CCS1477 and informed combination therapies that promote cancer cell killing.

Recurrent mutations in epigenetic regulators occur in >50% of patients with AML, including in *DNMT3A* and *TET2* (*6*–*13*). These mutant alleles are acquired early in AML progression and have prognostic, biologic, and therapeutic relevance in AML. DNMT3A and TET2 antagonistically regulate DNA methylation at CpG dinucleotides, such that loss-of-function mutations in these genes, as commonly found in AML, result in genome-wide hypo- or hypermethylation, respectively, and are required to maintain leukemogenesis (*14*–*19*). Although the US Food and Drug Administration–approved nucleoside analogs azacitidine and decitabine are considered hypomethylating agents (HMAs), the presence of *DNMT3A* or *TET2* mutations does not always portend therapeutic responsiveness, suggesting that other epigenetic or signaling mechanisms may be at play (*20*–*26*). *DNMT3A* and *TET2* mutations almost exclusively co-occur with additional disease alleles to induce leukemogenesis (*11*, *12*)*.* Constitutive activating mutations in the receptor tyrosine kinase *FLT3*, commonly internal tandem duplications (*ITD*), result in ligand-independent receptor activation and frequently co-occur with *DNMT3A* and *TET2* mutations (*8*, *12*, *27*–*31*). We and others have shown that *Dnmt3a* and/or *Tet2* somatic mutations or loss-of-function alleles combined with *Flt3*^*ITD*^ result in lethal AML in mice with similar epigenetic, transcriptional, and pathologic features as the human disease (*32*–*34*). Although the specific pathways epigenetically controlled by DNMT3A and TET2 remain poorly understood, we and others have shown that the altered DNA methylation and gene expression changes mediated by their loss of function are important for AML development and maintenance (*32*, *33*, *35*–*37*). Given this, we examined whether inhibition of broad transcriptional coactivators p300/CBP could counteract epigenetic dysregulation in *DNMT3A-* and *TET2*-mutant AML through conserved or distinct sets of pathways to suppress leukemogenesis. Further, in this study, we identify a multiagent approach to eradicate leukemia in mouse models of epigenetic-mutant AML.

## RESULTS

### *Tet2/Flt3*-mutant mice have accelerated leukemogenesis compared to *Dnmt3a/Flt3*-mutant mice

To directly compare and contrast *Flt3*^*ITD*^ AML harboring loss-of-function alleles of the opposing epigenetic modifiers *DNMT3A* and *TET2*, we used our previously published mouse model combining the somatic loss of *Dnmt3a* (*Dnmt3a^fl/fl^ Mx1-Cre*) (*38*, *39*) with germline knock-in of *Flt3*^*ITD*^ (*D/F*) (*32*, *40*) and generated mice with somatic loss of *Tet2* (*Tet2^fl/fl^ Mx1-Cre*) (*39*, *41*) and matching *Flt3*^*ITD*^ alleles (*T/F*) (*40*) (fig. S1A). Relying just on leaky *Mx1-Cre* expression without the use of pIpC to delete *Dnmt3a* and *Tet2* floxed alleles as we previously reported (fig. S1B) (*32*), we followed the mice for leukemia development and lethality. At moribundity, *D/F* and *T/F* mice exhibited significantly increased white blood cell counts in the peripheral blood (fig. S1C). Moribund *D/F* and *T/F* and, to a lesser, extent age-matched *D/F* mice exhibited splenomegaly associated with increased myeloid and decreased lymphoid (CD3 and B220) content as compared to wild-type (WT) controls (fig. S1, D and E). Moribund *D/F* and *T/F* mouse bone marrow contained increased myeloid populations by flow cytometry (fig. S1F). Histopathologic analyses showed greater than 20% immature forms/blasts in both the bone marrow and spleen, with age-matched *D/F* featuring an intermediate phenotype, as compared to age-matched WT controls (fig. S1G). This was supported by significantly increased lineage^−^ c-Kit^+^ Sca1^+^ (LSK) cells in the bone marrow and spleen by flow cytometry in *T/F* mice (fig. S1H). These data are consistent with our previous report describing the *D/F* AML mouse model (*32*). When compared to *D/F* mice, *T/F* mice have significantly shortened overall survival (OS; *D/F* median of 43 days versus *T/F* median of 24 days), which was maintained upon secondary transplantation of AML cells into WT recipients (fig. S1, I and J). Collectively, these pathologic and immunophenotypic features, along with the presence of greater than 20% immature forms/blast in the bone marrow and rapid lethality, are concordant with an AML diagnosis (*42*) and are consistent with other reports of similar mouse models (*33*, *34*). These data indicate that *Tet2* loss combined with *Flt3*^*ITD*^ results in an earlier-onset, more aggressive AML as compared to *Flt3*^*ITD*^ with *Dnmt3a* loss (*34*, *36*).

### p300/CBP inhibition extends survival of *Dnmt3a/Flt3* and *Tet2/Flt3* mutant AML mice

**Fig. 1.**
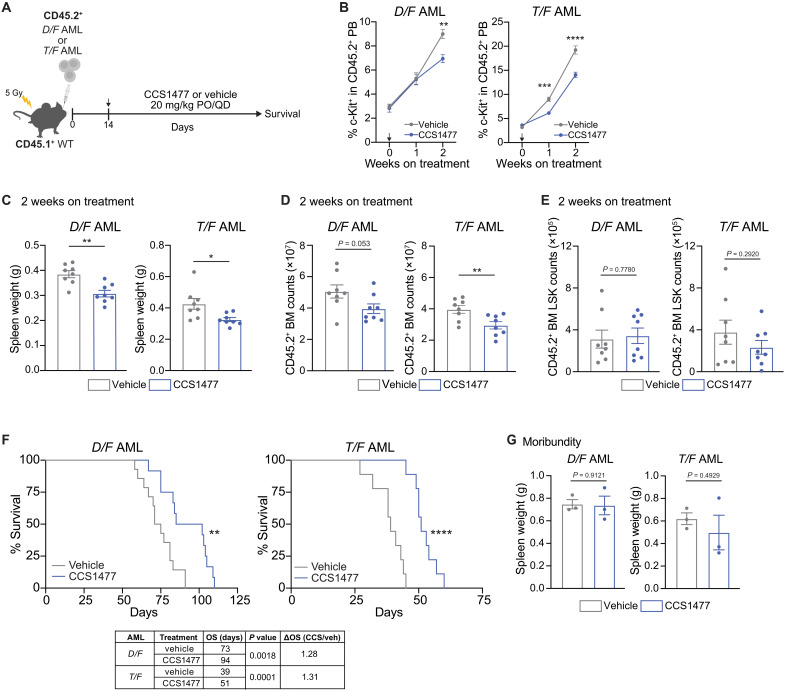
CCS1477 reduces leukemic burden in *Dnmt3a*/*Flt3*- and *Tet2/Flt3-*mutant mice. (**A**) In vivo CCS1477 treatment schematic for survival analyses. CD45.2^+^ *D/F* or *T/F* AML cells were transplanted into sublethally irradiated CD45.1^+^ WT recipient mice. Treatment was initiated 14 days posttransplant. CCS1477 (20 mg/kg) or vehicle was dosed by mouth (PO) once daily (QD). (**B**) Average ± SEM percent c-Kit^+^ cells in the CD45.2^+^ peripheral blood (PB) of CCS1477- and vehicle-treated *D/F* (*n* = 7 per treatment) and *T/F* (*n* = 11 per treatment) transplanted AML mice over 2 weeks. Week 0 time point was immediately before the first treatment, followed by weeks 1 and 2 on treatment. Significant differences were evaluated by two-way analysis of variance (ANOVA) Šídák’s multiple comparisons test. Average ± SEM (**C**) spleen weight, and numbers of (**D**) CD45.2^+^ and (**E**) CD45.2^+^ LSK (Lin^−^Sca1^+^Kit^+^) bone marrow cells of *D/F* (*n* = 8 per treatment) or *T/F* (*n* = 8 per treatment) AML mice after 2 weeks of CCS1477 or vehicle. Significant differences were evaluated by unpaired *t* test. (**F**) Kaplan-Meier survival analysis of CCS1477-treated and vehicle-treated *D/F* AML (CCS1477, *n* = 12; vehicle, *n* = 14) and *T/F* AML (CCS1477, *n* = 9; vehicle, *n* = 9) transplant recipient mice. Overall survival (OS) of moribund *D/F* AML mice was 94 and 73 days for CCS1477 and vehicle, respectively. OS of moribund *T/F* AML mice was 51 and 39 days for CCS1477 and vehicle, respectively. Significance determined by the log-rank (Mantel-Cox) test. (**G**) Average ± SEM spleen weight at mouse moribundity of CCS1477- or vehicle-treated *D/F* (*n* = 3 per treatment) or *T/F* (*n* = 3 per treatment) AML mice. Significant differences were evaluated by unpaired *t* test. Individual data points represent biological replicates. For all panels, *P < 0.05, **P < 0.01, ***P < 0.001, and ****P < 0.0001.

We next evaluated the efficacy of p300/CBP bromodomain inhibition by CCS1477 monotherapy in mice with AML. CD45.2^+^ *D/F* and *T/F* AML cells were transplanted into sublethally irradiated CD45.1^+^ WT recipient mice. Two weeks posttransplant, the CD45.2^+^ peripheral blood chimerism reached about 70%, at which point mice were treated with CCS1477 (20 mg/kg) (*4*, *5*) or vehicle control (Fig. 1A). CCS1477 treatment significantly slowed the expansion of circulating *D/F* and *T/F* leukemic blasts (CD45.2^+^ c-Kit^+^) compared to vehicle control treated mice (Fig. 1B), which was associated with significantly lower spleen weights and CD45.2^+^ leukemia cells in the bone marrow of mice after just 2 weeks on treatment (Fig. 1, C and D). We previously defined immunophenotypic LT-HSC (CD150^+^CD48^−^CD135^−^CD34^−^LSK) within the LSK compartment as the functional leukemia stem cell (LSC) in *D/F* AML mice (*43*). Despite the reduction of leukemic blasts within the peripheral blood, CCS1477 did not significantly lower LSK cells within the bone marrow (Fig. 1E). Following the mice until moribundity revealed that CCS1477 significantly prolonged survival compared to vehicle control treated mice (Fig. 1F). These data indicate that p300/CBP activity is important for disease progression of both *Dnmt3a/Flt3* and *Tet2/Flt3* mutant AML. However, both groups succumb to AML while on treatment, and, at the time of moribundity, both treatment groups exhibited similar splenomegaly, a marker of leukemic burden (Fig. 1G). These data suggest that p300/CBP inhibition impairs the proliferative capacity of *D/F* and *T/F* AML in vivo but is not curative as a monotherapy agent.

### CCS1477 impairs similar transcriptional programs in *Dnmt3a-* and *Tet2*-mutant leukemias

**Fig. 2.**
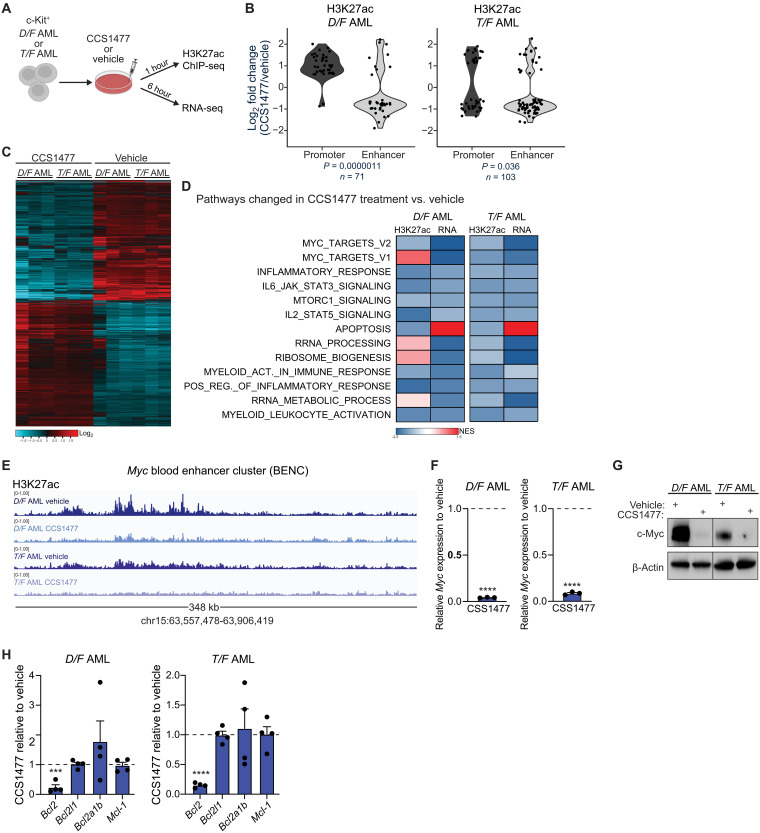
p300/CBP bromodomain inhibition alters enhancer activation and associated transcriptional programs. (**A**) Schematic of in vitro CCS1477 or vehicle treatments for H3K27ac chromatin immunoprecipitation sequencing (ChIP-seq) and RNA sequencing (RNA-seq). c-Kit^+^ splenocytes from moribund *D/F* or *T/F* AML mice were treated for 1 or 6 hours before ChIP-seq or RNA-seq, respectively. (**B**) Quantification of log_2_ fold change (adjusted p < 0.05) of H3K27ac at promoter and enhancer regions in CCS1477 versus vehicle in vitro–treated *D/F* (*n* = 2 per treatment) and *T/F* (*n* = 2 per treatment) c-Kit^+^ AML cells after 1 hour. Statistical differences were evaluated by Wilcoxon test. (**C**) Heatmap of differentially expressed genes (cut-off log_2_ fold change <−1, adjusted p < 0.05) by RNA-seq of c-Kit^+^ AML *D/F* (*n* = 3 per treatment) and *T/F* (*n* = 3 per treatment) treated in vitro with CCS1477 or vehicle. (**D**) Heatmap depiction of gene set enrichment analyses (GSEA) pathways in common by H3K27Ac ChIP-seq and RNA-seq in CCS1477- versus vehicle-treated *D/F* (left) and *T/F* (right) AML cells. Color corresponds to GSEA normalized enrichment score (NES). (**E**) Representative H3K27ac ChIP-seq tracks at *Myc* blood enhancer cluster (BENC). (**F**) Average ± SEM *Myc* expression by RT-qPCR in CCS1477 versus vehicle in vitro–treated *D/F* (*n* = 3 per treatment) and *T/F* (*n* = 3 per treatment) c-Kit^+^ AML cells after 6 hours. Significance determined by unpaired *t* tests. (**G**) Representative Western blot image for c-Myc in *D/F* and *T/F* AML cells after in vitro treatment of CCS1477 or vehicle after 6 hours. (**H**) Average ± SEM expression of apoptosis pathway genes by RT-qPCR in CCS1477- versus vehicle in vitro–treated *D/F* (*n* = 4 per treatment) and *T/F* (*n* = 4 per treatment) c-Kit^+^ AML cells after 6 hours. Significance determined by one-way ANOVA with Tukey’s multiple comparisons test. Individual data points represent biological replicates. For all panels, ***P < 0.001 and ****P < 0.0001.

To identify potential pathways that could be combinatorially targeted to improve treatment efficacy with CCS1477, we compared the mechanism(s) of p300/CBP-regulated epigenetic programs in *D/F* and *T/F* AML cells. Given that acetylation of H3K27 is solely performed by p300/CBP and that H3K27ac is a critical mark of active enhancers, we evaluated the effects of CCS1477 on H3K27 acetylation at enhancers and gene promoters in AML cells. Using in vitro doses defined by dose-titration cell viability assays (fig. S2A), CCS1477 caused rapid (within 1 hour) but transient reductions in global H3K27ac levels in *DNMT3A*-mutant human cell lines OCI-AML2 and OCI-AML3, as well as in *D/F* and *T/F* mouse AML (fig. S2B). At the nucleotide level, after 1 hour of CCS1477 treatment, genome-wide H3K27ac chromatin immunoprecipitation sequencing (ChIP-seq) identified significant reductions in H3K27ac at enhancer regions in both *D/F* and *T/F* AML cells (Fig. 2, A and B). We also detected changes in H3K27ac at promoters with CCS1477 treatment (Fig. 2B), which was unexpected given that p300 is predominantly found at intronic and intergenic regions (*5*, *44*). At the gene-level, CCS1477-mediated changes in H3K27ac were largely nonoverlapping between promoter and enhancer regions within the same AML genotype and were also distinct when comparing *D/F* AML versus *T/F* AML (fig. S2, C and D). However, despite the genotype specific patterns in H3K27ac (fig. S2D), we observed a conserved p300/CBP-regulated gene expression signature in both AML models by RNA sequencing (RNA-seq) (Fig. 2, A and C; fig. S2E; and data S1). Interrogation of H3K27ac and gene expression at the pathway level by gene set enrichment analysis (GSEA) revealed consistent inhibition of cancer cell growth–related pathways including MYC target genes, FLT3 signaling including JAK/STAT, and immune cell signaling and function by CCS1477 regardless of AML genotype (Fig. 2D and data S1). These results are consistent with the slowed leukemic expansion by CCS1477 monotherapy in vivo.

To further explore this link between p300/CBP-regulated genes and AML growth suppression, a closer look at the ChIP-seq data revealed a partial to near complete loss of H3K27ac at the specialized blood enhancer cluster (BENC) known to critically regulate *MYC* expression in normal and malignant hematopoietic stem cells (Fig. 2E) (*45*). Consistent with loss of H3K27ac at the BENC, *Myc* mRNA and c-Myc protein expression was also significantly diminished by CCS1477 (Fig. 2, F and G). Myc repression is likely contributing to the inhibition of AML proliferation and induction of cell cycle arrest (fig. S2, F and G) and may also explain the decreased ribosomal biogenesis and metabolism gene signatures associated with CCS1477 treatment (Fig. 2D). Although increased H3K27ac enriched for apoptosis-associated genes, this failed to translate to increased expression of apoptotic gene signatures or a significant induction of AML cell death by CCS1477 (Fig. 2D; fig. S2, H and I; and data S1). CCS1477 significantly down-regulated the prosurvival gene *Bcl2*, but expression of *Bcl2l1*, *Bcl2a1b*, and *Mcl-1* was unaltered (Fig. 2H). Collectively, these data explain why CCS1477 monotherapy impairs leukemia cell proliferation in AML and suggest that combination of CCS1477 with drugs that target cell survival pathways may be effective.

### Combination of p300/CBP, BCL2, and FLT3 inhibition targets AML stem/progenitors

**Fig. 3.**
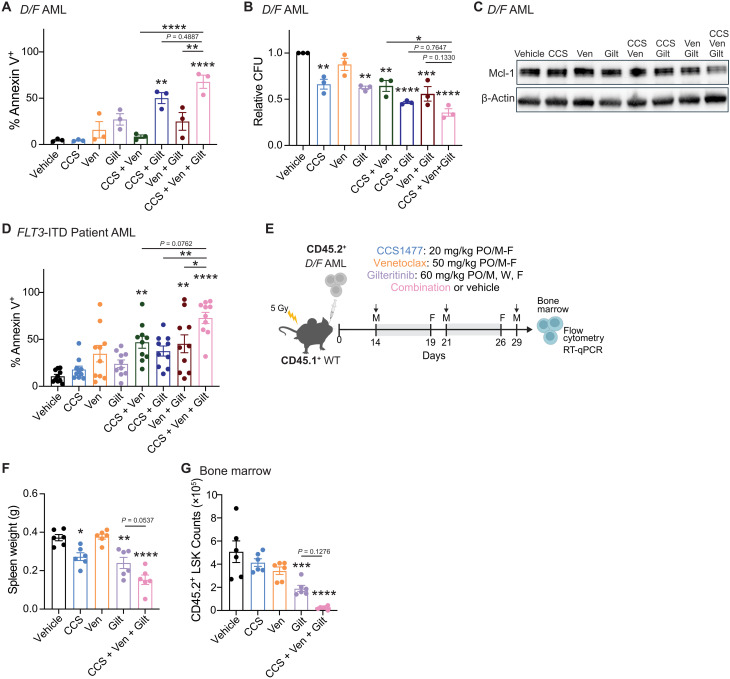
The triplet combination of CCS1477 with venetoclax and gilteritinib reduces leukemic burden in *Dnmt3a*/*Flt3* mutant-AML mice and *FLT3*-*ITD* patient AML. (**A**) Flow cytometric analyses of average ± SEM annexin V^+^ cells 3 days after in vitro treatment of CCS1477, venetoclax, gilteritinib, combination, or vehicle in c-Kit^+^ *D/F* AML cells (*n* = 3). Significance determined by one-way ANOVA with Tukey’s multiple comparisons test. (**B**) Average colonies [colony-forming units (CFU)] ± SEM formed by c-Kit^+^ *D/F* AML cells (*n* = 3) treated with CCS1477, venetoclax, gilteritinib, combination, or vehicle. Significance determined by one-way ANOVA with Tukey’s multiple comparisons test. (**C**) Representative Western blot image for Mcl-1 in *D/F* AML cells after in vitro treatment with CCS1477, venetoclax, gilteritinib, combination, or vehicle after 4 hours in the presence of proteasome inhibitor. (**D**) Average ± SEM annexin V^+^ cells by flow cytometric analyses after 3 days in vitro treatment of CCS1477, venetoclax, gilteritinib, combination, or vehicle in deidentified primary human *FLT3*-*ITD* AML patient samples (*n* = 10). Significance determined by one-way ANOVA with Tukey’s multiple comparisons test. (**E**) Schematic of in vivo combination therapy. Mice received either CCS1477 (*n* = 6), venetoclax (*n* = 6), gilteritinib (*n* = 6), combination (*n* = 6), or vehicle (*n* = 6) for 2 weeks. Mice were sacrificed 3 hours after the last treatment. Bar graphs of average ± SEM (**F**) spleen weight and (**G**) bone marrow CD45.2^+^ LSK (Lin^−^Sca1^+^Kit^+^) cells. Significance determined by one-way ANOVA with Tukey’s multiple comparisons test. Individual data points represent biological replicates. For all panels, *P < 0.05, **P < 0.01, ***P < 0.001, ****P < 0.0001.

To improve the efficacy of CCS1477, we devised a combinatorial approach to enhance cell death with venetoclax and gilteritinib. The selective BCL-2 inhibitor venetoclax is now part of a standard-of-care regimen in combination with HMAs or low-dose cytarabine for patients with newly diagnosed AML who are unfit for intensive chemotherapy (*46*). The second-generation type I FLT3 inhibitor gilteritinib is used routinely to treat *FLT3*-mutant relapse/refractory (R/R) AML (*47*). Moreover, preclinical studies combining venetoclax with FLT3 inhibitors, like gilteritinib, demonstrate synergistic induction of apoptosis through increased protein turnover of the anti-apoptotic factor MCL-1 (*48*–*50*). Given the co-occurrence of *FLT3* mutations with *DNMT3A* and *TET2* mutations, and our findings that *Bcl2*, but not *Mcl-1*, expression was reduced by CCS1477 (Fig. 2H) in the *FLT3*-mutant molecular subtype, we reasoned that the addition of venetoclax and gilteritinib would improve the therapeutic response of AML to CCS1477 by further tipping the balance toward cell death. The combination of CCS1477 with venetoclax and gilteritinib (CCS/ven/gilt) resulted in the most robust induction of apoptosis and reduction in clonogenicity of any single agent or double combination in *D/F* AML cells (Fig. 3, A and B). Although CCS1477 monotherapy does not result in transcriptional repression of *Mcl-1*, the CCS/ven/gilt triplet therapy did reduce Mcl-1 protein as compared to vehicle (Fig. 3C). We next treated *FLT3*-*ITD*–positive AML patient samples, many of which also harbor mutations in epigenetic modifiers (i.e., *DNMT3A*, *TET2*, *IDH1*, and *IDH2*) (table S1). The CCS/ven/gilt triplet therapy induced profound apoptosis of AML patient samples reaching greater than 50% in all but one case and produced significantly greater cell death compared to each monotherapy and even doublet combinations (Fig. 3D). Given these promising in vitro findings, we evaluated the CCS/ven/gilt triplet therapy in vivo using the *D/F* AML transplant model. Mice engrafted with *D/F* AML cells were treated with single agents CCS1477, venetoclax, gilteritinib, or the triple combination for 2 weeks (Fig. 3E). No significant changes in mouse weights were observed over the course of treatment, indicating that the triplet therapy was well tolerated (fig. S3A). At the end of the treatment period, CCS/ven/gilt provided the greatest decrease in spleen weights (Fig. 3F). Despite no differences in total bone marrow cellularity compared to vehicle (fig. S3B), immunophenotypic analyses revealed a significant reduction in leukemic progenitors [Lin^−^c-Kit^+^ (LK)] and almost complete eradication of LSC-enriched LSK cells within the bone marrow of CCS/ven/gilt-treated mice (fig. S3C and Fig. 3G). The average total number of immunophenotypic LSK remaining in CCS/ven/gilt-treated mice was ~10-fold lower (average ± SEM, 22,723 ± 5832 cells) than the next most effective treatment gilteritinib (average ± SEM, 190,862 ± 26,037 cells), demonstrating the superiority of this combination in targeting LSCs (Fig. 3G). Notably, the depletion of leukemic stem/progenitors by CCS/ven/gilt triplet therapy was not an artifact of transcriptional repression of *Kit* and *Sca1* because CCS1477 alone did not significantly affect the number of immunophenotypic AML LK or LSK cells (fig. S3C and Fig. 3G) despite decreases in mRNA transcript levels of these markers (fig. S3, D and E). CCS/ven/gilt triplet therapy also significantly induced myeloid maturation of AML cells in the bone marrow (fig. S3F) and is consistent with previous reports of gilteritinib promoting differentiation of *FLT3*-*ITD* AML (*51*). The decreased expression of putative p300-regulated genes *Bcl2*, *Flt3*, and *Myc* was greatest in the CCS/ven/gilt-treated group, albeit not significantly different from CCS1477 alone, suggesting that venetoclax and gilteritinib may provide an additive effect (fig. S3, G to I). Together, these data demonstrate that the combination of CCS1477 with venetoclax and gilteritinib is effective against primitive AML stem/progenitor cells. Our data suggest that mechanistically CCS/ven/gilt may cooperatively converge on multiple crucial progrowth and survival pathways.

## DISCUSSION

The transcriptional coactivators p300/CBP are attractive therapeutic targets in AML and other cancers, and the development of selective and potent p300/CBP bromodomain inhibitors [CCS1477 and FT7051; (*52*)] has demonstrated promising activity in preclinical and clinical settings. In this study, we evaluated p300/CBP inhibition by CCS1477 in two physiologically relevant *Flt3^ITD^* murine models with loss of either *Dnmt3a* or *Tet2*, representative of common subtypes of human AML. While CCS1477 caused unique changes in H3K27ac depending on which epigenetic modifier was mutated, the transcriptional signatures and biologic consequences of CCS1477 were highly similar across these AML models. This was unexpected given the opposing activities of DNMT3A and TET2 and the distinct DNA methylation profiles in mouse and human AML with loss-of-function mutations in these genes. Consistent with previous preclinical studies including other subtypes of AML such as those with *MLL* rearrangements (*5*), we found that CCS1477 impaired leukemogenesis primarily through inhibiting cell proliferation. Expression of the oncogene *Myc* was also consistently reduced in our and other’s studies, but whether decreased Myc alone is responsible for disrupting cancer cell proliferation, or a combination of several p300/CBP regulated genes, has not been definitively shown. Collectively, studies demonstrate that p300/CBP inhibition by CCS1477 slows tumor progression.

There is an immediate need for more specific and potent combinatorial regimens for the treatment of AML that are less toxic than the standard-of-care chemotherapies. A major clinical challenge to overcome therapy resistance and improve patient survival is the ability to target LSCs that are responsible for disease initiation and propagation through self-renewal properties. LSCs maintain phenotypic and epigenetic plasticity that enable therapy resistance by switching between dormant and proliferative states, metabolic reprogramming, and dysregulation of apoptotic pathways in addition to other mechanisms. In the case of venetoclax and azacitidine (ven/aza), this combination was shown to target LSCs in patients with AML by impairing oxidative phosphorylation and energy metabolism (*53*) yet is not curative due to acquired resistance, such as up-regulation of BCL-XL and MCL-1 (*54*). Preclinical studies in *FLT3* WT and mutant AML revealed venetoclax and gilteritinib (ven/gilt) induced apoptosis through MCL-1 suppression (*48*–*50*). In a phase 1b study, ven/gilt resulted in a high modified composite complete response, which was better than single-agent gilteritinib. Yet the OS remained comparable between those two studies (*47*, *55*). Recently, the addition of gilteritinib to ven/aza in a phase 1/2 study in patients with *FLT3*-mutated AML demonstrated encouraging tumor response rates and survival benefits (*56*), although the effects of this triplet combination on LSCs have yet to be elucidated.

Here, we describe another triplet combination comprising CCS/ven/gilt that profoundly induces AML cell death and targets LSCs. CCS/ven/gilt provided maximum suppression of pro-oncogenic survival and proliferation factors to effectively block leukemogenesis. CCS1477 alone resulted in decreased mRNA expression of a variety of known leukemia dependencies including *Kit*, *Myc*, *Flt3*, *Bcl2*, and others, but this was not enough to induce AML cell death. It was previously demonstrated that p300 acetylates MCL-1 at K40, protecting it from proteasomal degradation (*57*). In our studies, we were unable to detect increased turnover of MCL-1 with CCS1477 alone. Nonetheless, p300 regulation of MCL-1 turnover remains a possibility as, in our study, the CCS/ven/gilt triplet therapy caused the greatest reduction in MCL-1 protein levels, even compared to ven/gilt, and was associated with significant induction of apoptosis. Another oncogenic factor that appears to be combinatorially repressed with CCS/ven/gilt therapy is *MYC*. At the transcriptional level, we postulate that the additive repression of *MYC* by the triplet therapy is achieved by the direct regulation of the *MYC* BENC by p300/CBP inhibition and the inhibition of FLT3-mediated STAT5 activity also on the *MYC* enhancer (*58*, *59*). Thus, the therapeutic benefit of this triplet combination, at least in part, stems from its simultaneous repression of multiple pathways involved in leukemogenesis. However, the effects of diminished LSCs by CCS/ven/gilt on survival as compared to other emerging combinations, including ven/aza/gilt, need to be further investigated. We anticipate that CCS/ven/gilt offers unique advantages by repressing additional oncogenic pathways via transcription regulation. While the exact antileukemogenic molecular mechanisms behind CCS/ven/gilt, particularly targeting LSCs, remain to be fully elucidated, this work provides strong evidence for testing this triplet therapy in patients.

## MATERIALS AND METHODS

### Mice

*Flt3^ITD/ITD^* mice (*40*) were bred with *Dnmt3a^fl/fl^* mice (*38*) or *Tet2^fl/fl^* mice (*41*) with *Mx1-Cre* (*39*) to generate *Flt3^ITD/ITD^Dnmt3a^fl/fl^Mx1-Cre* (referred to as *D/F*) mice (*32*) and *Flt3^ITD/ITD^ Tet2^fl/fl^Mx1-Cre* (referred to as *T/F*) mice. *Mx1-Cre* was maintained as heterozygous in all crosses. As previously reported (*32*), *D/F* mice are not treated with pIpC, but, instead, hematopoietic cells are allowed to spontaneously delete one or both *Dnmt3a* alleles. *T/F* mice are also not treated with pIpC, allowing for spontaneous deletion of one or both *Tet2* alleles. All mice are maintained on a CD45.2^+^ C57BL6/J background. Age-matched WT C57BL6/J mice (Charles River Laboratories) or *D/F* mice were 22 to 28 days of age. For secondary transplants, CD45.1^+^ C57BL6/J mice (Charles River Laboratories), 7 to 8 weeks of age, were irradiated with 700 rads before tail vein injection of 1 million c-Kit^+^ AML cells from moribund *D/F* or *T/F* mice. Transplant AML experiments include *n* ≥ 2 donors per group. All mice were housed, bred, and used at Thomas Jefferson University in accordance with the approved Institutional Animal Care and Use Committee protocol (IACUC protocol no. 01932).

### In vivo treatments

For CCS1477 monotherapy in vivo studies, CD45.1^+^ C57BL6/J mice (Charles River Laboratories), 7 to 8 weeks of age, were irradiated with 500 rads before transplantation with either 10 million CD45.2^+^ *D/F* AML cells or 2.5 million CD45.2^+^ *T/F* AML cells via retro-orbital injection. Two weeks following transplantation, peripheral blood was assessed to ensure at least 70% donor CD45.2^+^ AML cells before treatment. Recipient mice were treated with CCS1477 (20 mg/kg; inobrodib, CellCentric) once daily or vehicle [0.5% methylcellulose and 10% dimethyl sulfoxide (DMSO)]. For survival analyses, mice were treated once daily until moribundity. A separate cohort of mice were treated daily only for 2 weeks and obtained weekly peripheral bleeds for flow cytometric analyses. At the end of 2 weeks, mice were euthanized, spleens were weighed, and bone marrow was collected for flow cytometric analyses.

For CCS1477 combination in vivo studies, CD45.1^+^ C57BL6/J mice (Charles River Laboratories), 7 to 8 weeks of age, were irradiated with 500 rads before transplantation with 10 million CD45.2^+^ *D/F* AML cells via retro-orbital injection. Two weeks following transplantation, peripheral blood was assessed to ensure at least 70% donor CD45.2^+^ AML cells before treatment. Mice were randomized and treated with vehicle (0.5% methylcellulose and 10% DMSO), CCS1477 (CellCentric, 20 mg/kg daily Monday to Friday), venetoclax (Chemietek, 50 mg/kg daily Monday to Friday), gilteritinib (Chemietek, 60 mg/kg thrice weekly Monday, Wednesday, and Friday), or combination CCS1477, venetoclax, and gilteritinib (same dosage as single agents). All treatments were given by oral gavage for 2 weeks and prepared in 0.5% methylcellulose and 10% DMSO. Mice were euthanized at the end of treatment. Spleens were collected and weighed, and bone marrow was collected for flow cytometric analyses and reverse transcription quantitative polymerase chain reaction (RT-qPCR). Transplant AML experiments include *n* ≥ 2 donors per group.

### Histology and cytospins

Bone marrow and spleen were harvested from moribund *T/F* AML mice and age-matched WT and *D/F* mice. For histology, organs were fixed with paraformaldehyde, embedded in paraffin and stained with hematoxylin and eosin by the Sidney Kimmel Comprehensive Cancer Center (SKCCC) Translational Research/Pathology Core. Images were taken on Aperio Scanscope CS2 microscope and analyzed using ImageScope v12.4.6.5003. For cytospins, 0.1 × 10^6^ bone marrow cells, without red blood cell (RBC) lysis, were resuspended in cytospin buffer [1× Cell Dissociation buffer, 5% fetal bovine serum (FBS), and 2% bovine serum albumin]. Cells were spun onto premoistened Superfrost Plus Microscope slides in a single layer using the Cytospin 4 (Thermo Fisher Scientific). Slides were dried, fixed, and stained with Wright-Giemsa. Images were taken using EVOS FL Color Imaging (Invitrogen).

### Flow cytometric analyses and cell enrichments

Bone marrow or spleen cells were washed in 1× fluorescence-activated cell sorting (FACS) buffer [1% FBS and 0.01% sodium azide in phosphate buffered saline (PBS)] after RBC lysis. All antibodies were purchased from BioLegend unless otherwise stated. To stain stem/progenitor cell populations, single-cell suspensions were first stained with a cocktail of biotin-conjugated antibodies against CD3 (clone 145-2C11), CD4 (clone RM4-5), CD8 (clone 53-6.7), CD11b (clone M1/70), Gr1 (clone RB6-8C5), Ter119 (clone TER-119), CD45R (clone RA3-6B2), CD19 (clone 6D5), CD127 (clone A7R43). PE-Cy7–conjugated anti-Sca1 (clone D7), BV650-conjugated anti-CD117 (clone 2B8), PerCP-Cy5.5–conjugated anti-CD16/32 (clone 93), and fluorescein isothiocyanate–conjugated anti-CD34 (clone RAM34) antibodies were also used in this first stain. Cells were then washed and stained with streptavidin-conjugated APC-Cy7. For mature myeloid cell populations, cells were stained with BV421-conjugated anti-CD11b (clone M1/70) and PE-Cy7–conjugated anti-Gr1 (clone RB6-8C5) antibodies. For lymphoid cell populations, cells were stained with AF700-conjugated anti-CD45R/B220 (clone RA3-6B2) and APC-conjugated anti-CD3ε (clone 500A2) antibodies. For cell cycle analyses, cells were stained with Vybrant DyeCycle Violet Stain (Invitrogen) according to manufacturers’ instructions. For apoptosis analyses, cells were stained with PacBlue-conjugated annexin V (BioLegend) and propidium iodine (BioLegend) according to the manufacturer’s instructions. All flow cytometric analyses were performed on LSRFortessa (BD Biosciences), Symphony A5 (BD Biosciences), or Symphony A3 (BD Biosciences). Data analyses were performed using FlowJo software. Total cell counts for selected cell populations were calculated as follows: (gated population count/gated live-singlet count)*total cell count of mouse bone marrow or spleen.

c-Kit^+^ enrichments of bone marrow or spleen AML cells were performed using CD117 microbeads according to the manufacturer’s instructions (Miltenyi Biotec) using AutoMACS Pro separator or magnetic stand (Miltenyi Biotec).

### In vitro treatments

Splenocytes from moribund *D/F* or *T/F* AML mice were isolated, RBCs were lysed, and c-Kit^+^ cells were enriched. Cells were plated at a concentration of 0.125 × 10^6^ cells/ml in growth medium RPMI 1640 (Life Technologies) supplemented with 20% FBS (HyClone), 1% penicillin and streptomycin (Life Technologies), and recombinant mouse stem cell factor (SCF; 60 ng/ml), interleukin-6 (IL-6; 20 ng/ml), and IL-3 (20 ng/ml) (Miltenyi Biotec). Cells were treated with 4 μM or 2 μM CCS1477 for *D/F* or *T/F* AML, respectively, or DMSO, unless otherwise stated. For combination treatments, cells were treated with 0.5 μM CCS1477, 2 μM venetoclax, and 0.25 μM gilteritinib. All cells were incubated in a humidified environment at 37°C with 5% CO_2_ for 3 days before analyses, unless otherwise stated.

OCI-AML2 (DSMZ) and OCI-AML3 cells (DSMZ) were plated at a concentration of 0.125 × 10^6^ cells/ml in α–minimum essential medium plus nucleosides (Life Technologies) supplemented with 20% heat-inactivated FBS (Optima) and 1% penicillin and streptomycin (Life Technologies). Cells were treated with 250 nM CCS1477 or DMSO, unless otherwise stated. All cells were incubated in a humidified environment at 37°C with 5% CO_2_ for 3 days before analyses, unless otherwise stated.

### Drug dose-titration assays

Splenocytes from moribund *D/F* or *T/F* AML mice were isolated and c-Kit^+^ enriched. Mouse or human AML cells were plated in triplicate at 0.125 × 10^6^ cells/ml in cell growth medium as detailed above. CCS1477 was titrated and cells incubated for 3 days. Cell viability was determined using CellTiter Glo (Promega) according to the manufacturer’s instructions.

### Colony forming assays

A total of 1500 c-Kit^+^ leukemic splenocytes from moribund *D/F* or *T/F* AML mice were plated in triplicate in Methocult GF M3434. Cells were treated with varying doses of CCS1477 or DMSO. For combination therapy colony-forming-unit assays, cells were treated with 0.5 μM CCS1477, 2 μM venetoclax, and 0.25 μM gilteritinib or DMSO. Colonies were enumerated after 6 days. For human AML cell lines, 1500 OCI-AML2 or OCI-AML3 cells were plated in triplicate in Methocult H4435 and treated with either 50 nM CCS1477, 250 nM CCS1477, or DMSO. Colonies were enumerated after 7 days.

### Western blot analysis

Equal numbers of OCI-AML2 and OCI-AML3 cells were treated with 250 nM CCS1477 or DMSO and harvested after 1, 3, 6, 12, and 24 hours. Equal numbers of c-Kit^+^ leukemic splenocytes cells from moribund *D/F* or *T/F* AML mice were plated and treated with 4 μM or 2 μM CCS1477, respectively, or DMSO and harvested after 1, 3, 6, and 12 hours. For combination treatment, cells were treated with 0.5 μM CCS1477, 2 μM venetoclax, and 0.25 μM gilteritinib or DMSO for 4 hours in the presence of 2 μM MG-132 (2194, Cell Signaling Technologies). Cells were washed in PBS and then lysed in RIPA buffer with 1× Halt phosphatase and protease inhibitor cocktail (Thermo Fisher Scientific). Equal amounts of protein lysates were separated on bis-tris 4 to 12% gradient gels (NuPAGE) and transferred to polyvinylidene difluoride (PVDF) membranes using an iBlot2 (Invitrogen) in iBlot 2 PVDF mini stacks (Invitrogen). Membranes were blocked in 5% milk in 1× TBST for 1 hour and then incubated with primary antibodies to detect: anti-histone H3K27ac (ab4729, Abcam), anti-histone H3 (ab1791, Abcam), anti–c-Myc (ab32072, Abcam), anti–Mcl-1 (5453, Cell Signaling Technologies), and anti–β-actin (A5441, Sigma-Aldrich). Secondary detection was used with HRP conjugated anti-rabbit or anti-mouse antibodies (Thermo Fisher Scientific) following ECL Western Blotting Substrate (Thermo Fisher Scientific) addition and exposure on autoradiographic film (Thomas Scientific) or Bio-Rad ChemiDoc MP Imaging System.

### RNA isolation, cDNA synthesis, and RT-qPCR

RNA was isolated by TRIzol extraction (Life Technologies) according to manufacturer’s protocol. cDNA was synthesized using the High-Capacity cDNA kit (Life Technologies). Gene expression was quantified on QuantStudio 3 by TaqMan (Thermo Fisher Scientific) qPCR using the delta-delta Ct method for *Dnmt3a* (Mm00432881_m1), *Tet2* (Mm01320358_m1), *Myc* (Mm00487804_m1), *Bcl2* (Mm00477631_m1), *Bcl2l1* (Mm00437783_m1), *Bcl2a1b (*Mm03646861_mH), *Mcl-1* (Mm01257351_g1), *Kit* (Mm00445212_m1), *Sca1* (Mm00726565_s1), and *Flt3* (Mm00439016_m1)*.* Mouse *Sdha* (Mm01352366_m1) served as the housekeeping control.

### ChIP-seq analysis

c-Kit^+^ splenocytes from moribund *D/F* or *T/F* AML mice were plated and treated with 4 μM or 2 μM CCS1477, respectively, or DMSO for 1 hour. Cells were cross-linked using 1% formaldehyde for 10 min, and the reaction was stopped by the addition of 0.125 M glycine. Cell pellets were washed twice in cold PBS. Nuclear preparation was performed using the truChIP Chromatin Shearing Kit (Covaris) according to the manufacturer’s instructions. Samples were transferred to a 1 ml of AFA fiber milliTUBE (Covaris), and nuclear lysates were sonicated using a S220 ultrasonicator (Covaris) for 5 min total [2 min on and 30 s off (×2), followed by 1 min on] at 200 cycles per burst (CPB), 5% duty factor (DF). Chromatin immunoprecipitation (ChIP) was carried out for 2 hours at 4°C in a rotation wheel at 20 rpm with 30 μl of Dynabeads M-280 (Invitrogen) previously conjugated with 4 μg of anti-H3K27ac (ab4729, Abcam) antibody.

Beads were washed twice with low-salt washing buffer [150 mM NaCl, 0.5% Na deoxycholate, 0.1% NP-40, 1 mM EDTA (pH 8.0), and 50 mM tris-HCl (pH 8.0)], once with high-salt washing buffer [500 mM NaCl, 0.5% Na deoxycholate, 0.1% SDS, 1% NP-40, 1 mM EDTA (pH 8.0), and 50 mM tris-HCl (pH 8.0)], twice with LiCl washing buffer [250 mM LiCl, 0.5% Na deoxycholate, 0.1% SDS, 1% NP-40, 1 mM EDTA (pH 8.0), and 50 mM tris-HCl (pH 8.0)], and lastly washed twice with TE buffer [0.25 mM EDTA (pH 8.0) and 10 mM tris-HCl (pH 8.0)]. Next, ChIP-bound fractions were extracted using an elution buffer (1% SDS and 100 mM NaHCO_3_) and reverse cross-linked using 0.3 M NaCl with ribonuclease A (10 mg/ml) at 37°C for 30 min. DNA was incubated with proteinase K (0.2 mg/ml) overnight at 65°C. Then, DNA was extracted using a QIAquick PCR purification kit (QIAGEN), and DNA was eluted in 30 μl of 10 mM tris-HCl (pH 8.5). Sequencing was performed at 25 million reads per sample.

Paired-end FASTQ files were aligned to the mouse reference genome (GRCm38, Ensembl) using Chromap with the preset “chip” option. SAM-to-BAM conversion, sorting, and indexing were performed with samtools. Reads overlapping blacklist regions from iGenomes were removed with bedtools intersect. For signal visualization, normalized coverage tracks (bigWig) were generated from filtered BAM files using bedtools genomecov and scaled to 1 million mapped reads per sample. Tracks were sorted and converted to bigWig format using UCSC tools bedSort and bedGraphToBigWig. Peak calling was performed using MACS2 with matched input controls, using default parameters and a *q*-value threshold of 0.01. Peak annotation was conducted using HOMER. Peaks across conditions were merged using mergePeaks. Quantification of ChIP-seq signal over merged peaks was conducted using featureCounts (Rsubread). Differentially enriched peaks were determined using DESeq2. Peaks were classified as promoter-proximal or enhancer-associated based on distance to the nearest transcriptional start site (TSS; ±1000 base pairs). All plots were generated using ggplot2 and ggpubrin R. To create the preranked list for GSEAPreranked v4.4.0 analyses (*60*, *61*), all annotated peaks associated with genes were assigned a rank metric by calculating the −log_10_(*P* value)*sign(fold change). Raw (nominal) *P* values were used. For genes with multiple peaks, the gene peak with the highest rank metric was used.

### RNA-seq analysis

c-Kit^+^ splenocytes from moribund *D/F* or *T/F* AML mice were plated and treated with 4 μM or 2 μM CCS1477, respectively, or DMSO for 6 hours. RNA was purified by TRIzol extraction (Life Technologies). cDNA libraries were sequenced at 30 million reads per sample. Data were processed and differential gene expression analyses performed using AltAnalyze 2.1.3 (*32*, *62*). The expression output file was used to generate a preranked gene list wherein all expressed genes were ranked by −log_10_(*P* value)*sign(fold change) and then analyzed using GSEAPreranked v4.4.0 (*60*, *61*).

### Patient sample collection and treatment

*FLT3*-*ITD* human AML patient bone marrow and peripheral blood specimens were collected with written informed consent at Thomas Jefferson University Hospital. Studies were conducted in accordance with Institutional Review Board–approved protocols (IRB protocol no. 17D.083) and the US 2018 Common Rule on de-identified specimens and/or clinical data. Ficoll-purified mononuclear cells from 10 independent *FLT3*-*ITD* patients (table S1) were thawed and plated at a concentration of 0.125 × 10^6^ cells/ml in growth medium RPMI 1640 (Life Technologies) supplemented with 20% FBS (HyClone), 1% penicillin and streptomycin (Life Technologies), and recombinant human SCF (60 ng/ml), IL-6 (20 ng/ml), IL-3 (20 ng/ml), thrombopoietin (TPO; 20 ng/ml), and FLT3 ligand (20 ng/ml) (Miltenyi Biotec). Cells were allowed to recover overnight, then were replated as the day before, and treated with 0.5 μM CCS1477, 2 μM venetoclax, and/or 0.25 μM gilteritinib for 3 days before flow cytometric analyses.

### Statistics

Statistical analyses were performed using Prism 10.2.3. Statistical tests performed, as appropriate, include log-rank (Mantel-Cox) test of Kaplan-Meier survival curves, unpaired *t* tests, ordinary one-way analysis of variance (ANOVA), or two-way ANOVA with multiple comparisons tests as needed. See the figure legends for details.

## Supplementary Materials

### The PDF file includes:

Figs. S1 to S3 Table S1 Legend for data S1 Uncropped Western blots
