# Supplementary material for: P300/CBP inhibition with inobrodib in combination with gilteritinib and venetoclax targets leukemia stem cells in epigenetic mutant AML
Source: Sci Adv. 2026 May 15;12(20):eaec9305. doi: 10.1126/sciadv.aec9305 (PMC13178526; doi:10.1126/sciadv.aec9305)

Science Advances
AAAS

Supplementary Materials for

P300/CBP inhibition with inobrodib in combination with gilteritinib and venetoclax targets leukemia stem cells in epigenetic mutant AML

Melanie L. Goetz *et al.*

Corresponding author: Sara E. Meyer, sara.meyer@jefferson.edu

Sci. Adv. **12**, eaec9305 (2026)
DOI: 10.1126/sciadv.aec9305

The PDF file includes:

Figs. S1 to S3
Table S1
Legend for data S1
Uncropped Western blots

Other Supplementary Material for this manuscript includes the following:

Data S1

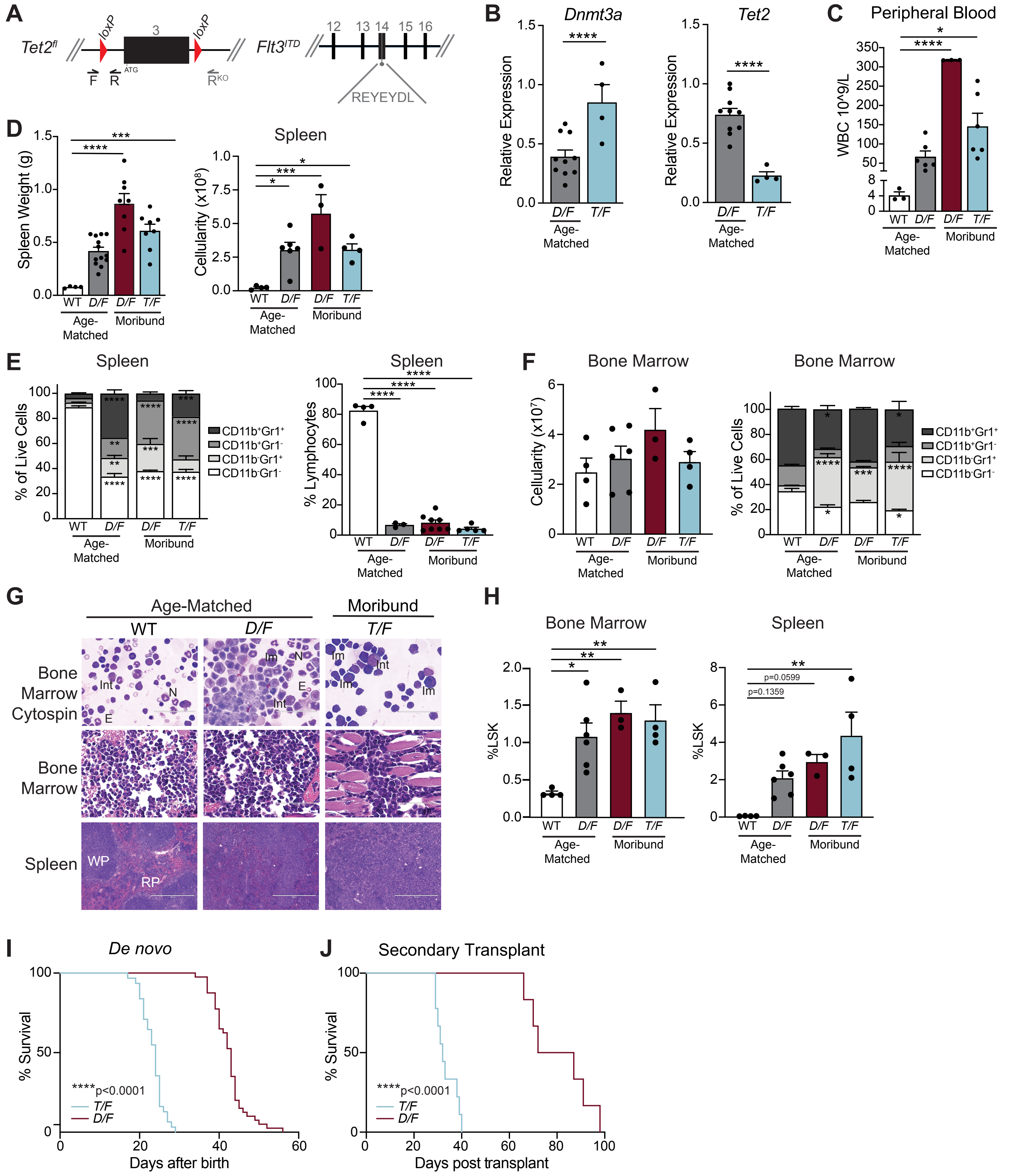

Fig. S1. Mice with *Tet2* deletion and *Flt3*^{ITD} develop AML. (A) Schematic of mouse *Tet2* exon 3 flanked by *LoxP* sites (*Tet2*^{fl}) and ITD knock-in to *Flt3* (*Flt3*^{ITD}). **(B)** Average relative expression ±SEM of *Dnmt3a* and *Tet2*

in c-Kit+ AML from moribund *T/F* (n=4) and age-matched *D/F* (n=10) mice. Statistical significance was determined by unpaired t-test. **(C)** Average ±SEM WBC count in peripheral blood from age-matched WT (n=3) and *D/F* (n=6) mice, and moribund *D/F* (n=3) and *T/F* (n=6) AML mice. Statistical significance was determined by one-way ANOVA with Tukey's multiple comparisons test. **(D)** Average ±SEM spleen weights of age matched WT (n=4) and *D/F* (n=13) mice and moribund *D/F* (n=8) and *T/F* (n=8) AML mice. Average ±SEM total spleen cellularity of age matched WT (n=4) and *D/F* (n=6) mice and moribund *D/F* (n=3) and *T/F* (n=4) AML mice. Significance determined by one-way ANOVA with Tukey's multiple comparisons test. **(E)** Average ±SEM proportion of myeloid (CD11b, Gr1) and lymphoid (CD3, B220) in the spleens of age-matched WT (n=4) and *D/F* (n=8) mice, and moribund *D/F* (n=3) and *T/F* (n=5) AML mice. Significant differences were evaluated by 2-way ANOVA Šídák's multiple comparisons test or one- way ANOVA with Tukey's multiple comparisons test for myeloid or lymphoid population analysis, respectively. **(F)** Average ±SEM total bone marrow cells and proportion of myeloid cells in the bone marrow of age-matched WT (n=4) and *D/F* (n=8) mice, and moribund *D/F* (n=3) and *T/F* (n=5) AML mice. No significant differences were determined in bone marrow cellularity by one-way ANOVA with Tukey's multiple comparisons. Significant differences in bone marrow myeloid cell populations were determined by two-way ANOVA Šídák's multiple comparisons test. **(G)** Representative Wright Giemsa-stained bone marrow cytospins and hematoxylin and eosin (H&E)-stained bone marrow and spleen from age-matched WT and *D/F* mice and moribund *T/F* AML mice. Bone marrow of *T/F* AML samples show a loss of erythroid (E) and myeloid cells including neutrophils (N) and intermediate myeloid cells (Int) and gain the presence of immature (Im) forms. *T/F* AML spleen shows loss of normal red pulp (RP) and gain of blast cell containing white pulp (WP). **(H)** Average ±SEM proportion of LSK ($Lin^{-}Sca1^{+}Kit^{+}$) in bone marrow and spleen from age-matched WT (n=4) and *D/F* (n=6) mice, and moribund *D/F* (n=3) and *T/F* (n=4) AML mice. Significance determined by one-way ANOVA with Tukey's multiple comparisons test. **(I)** Survival curves of transgenic *D/F* (n=40) and *T/F* (n=31) *de novo* AML mice. **(J)** Survival analysis of recipient mice transplanted with c-Kit+ *D/F* (n=6) or *T/F* (n=9) AML. Statistical significance was determined by log-rank (Mantel-Cox) test. Individual data points represent biological replicates. ****p<0.0001, ***p<0.001, **p<0.01, *p<0.05.

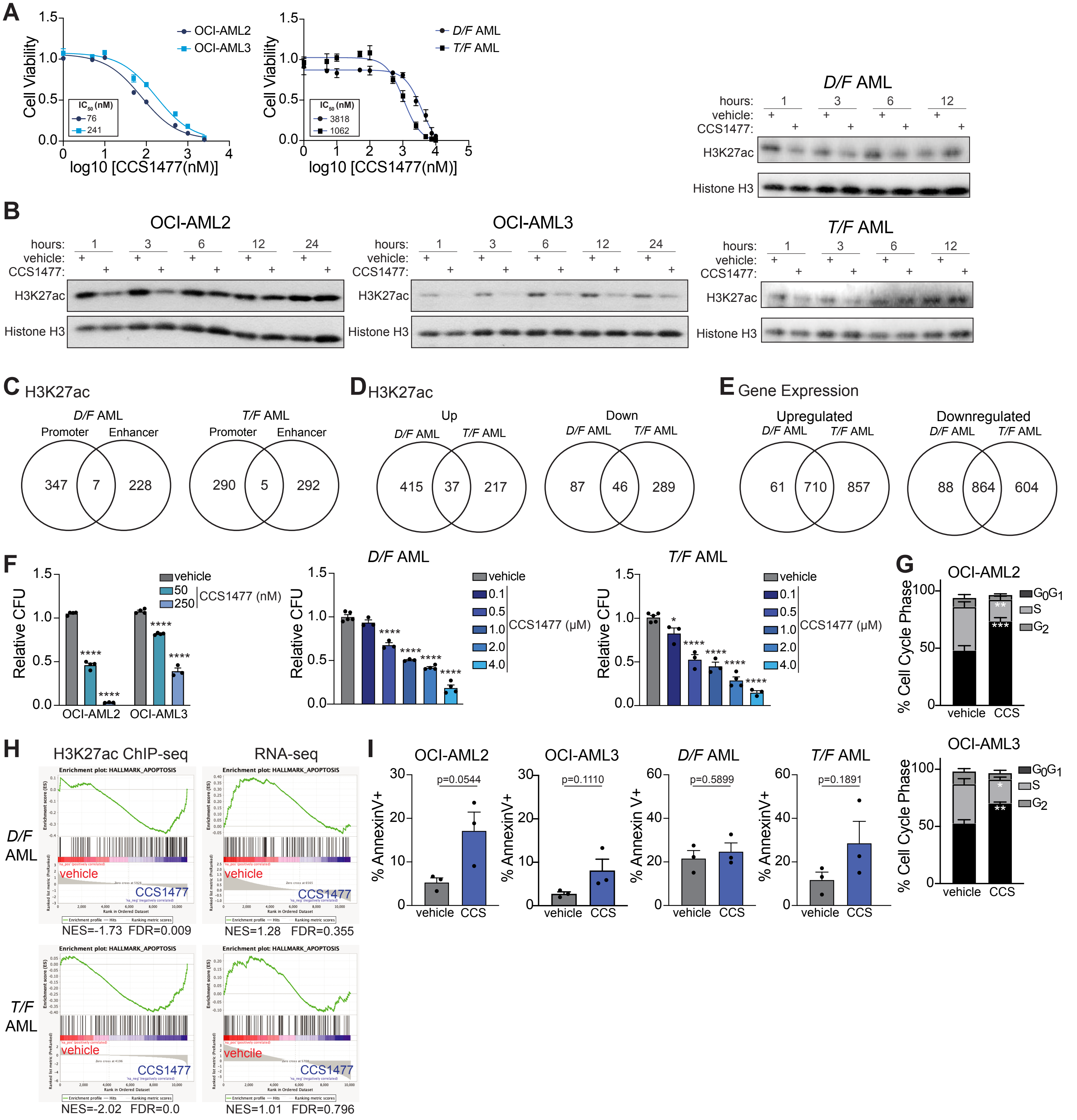

Fig. S2. CCS1477 alters histone acetylation and gene expression to impair the proliferation of leukemia cells. (A) *In vitro* dose-titration of CCS1477 on human OCI-AML2 (n=3), OCI-AML3 (n=3), and mouse c-Kit+ *D/F* (n=3) and *T/F* (n=3) AML cells after three days. **(B)** Representative western blot images of H3K27ac in OCI-AML2 and OCI-AML3 cells treated with CCS1477 or vehicle for 1-24 hours, and c-Kit+ *D/F* and *T/F* AML cells treated for 1-12 hours. **(C)** Venn diagrams of H3K27ac peak associated genes in promoter versus enhancer regions in CCS1477 treated AML by ChIP-seq (n=2). **(D)** Venn diagrams of up and down H3K27ac peak

associated genes in *D/F* (n=2) versus *T/F* (n=2) CCS1477 treated AML. **(E)** Venn diagrams of up- and down-regulated genes in CCS1477 treated *D/F* (n=3) versus *T/F* (n=3) AML by RNA-seq. **(F)** Average colonies ±SEM formed by OCI-AML2, OCI-AML3, and c-Kit$^+$ *D/F* and *T/F* AML cells treated with CCS1477 (n≥3/dose) or vehicle. Significant differences were evaluated by 2-way ANOVA Šídák's multiple comparisons test for human AML cells and by one-way ANOVA with Tukey's multiple comparisons test for mouse AML cells. **(G)** Proportion of OCI-AML2 (n=3) and OCI-AML3 (n=3) cells in each phase of cell cycle (average±SEM) after 3 days *in vitro* treatment with CCS1477 or vehicle. Significant differences were evaluated by 2-way ANOVA Šídák's multiple comparisons test. **(H)** Apoptosis GSEA enrichment plots of CCS1477 treated *D/F* and *T/F* AML cells compared to vehicle by H3K27ac ChIP-seq (n=2) and RNA-seq (n=3). **(I)** Average ±SEM AnnexinV$^+$ cells 3 days after *in vitro* dose of CCS1477 or vehicle in OCI-AML2 (n=3) cells, OCI-AML3 (n=3) cells, and c-Kit$^+$ *D/F* (n=3) or *T/F* (n=3) AML cells. No significant differences were determined by unpaired t-test. Individual data points represent biological replicates. ****$p<0.0001$, **$p<0.01$, *$p<0.05$.

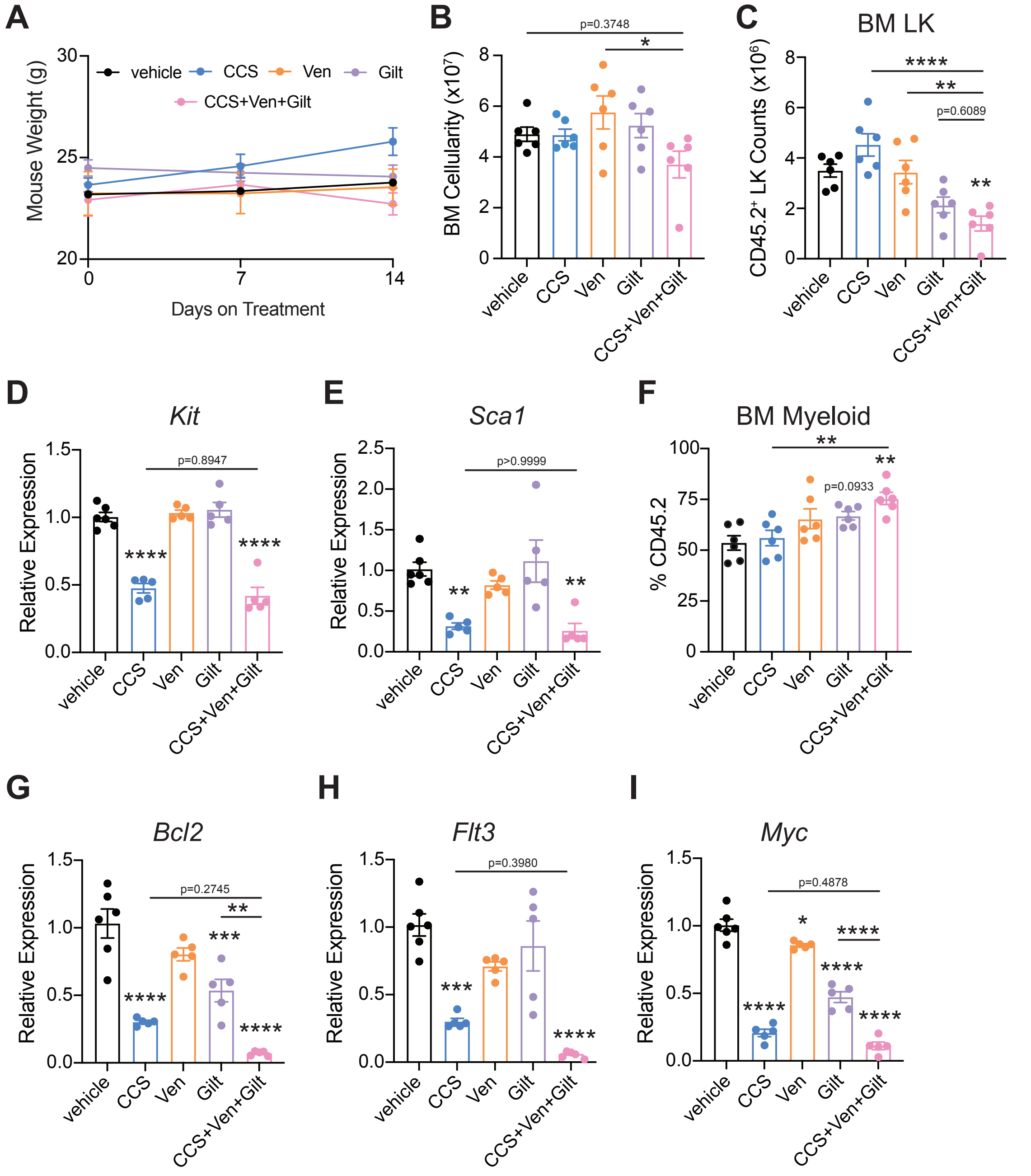

Fig. S3. Combined inhibition of p300/CBP, BCL2, and FLT3 is an effective and novel therapeutic strategy for *Dnmt3a/Flt3*-mutant AML. (A) Average mouse weights over two weeks of treatment (n=6/treatment). No significant differences were determined by 2-way ANOVA Šídák's multiple comparisons test. Average ±SEM **(B)** total and **(C)** CD45.2^{+} LK (Lin^{-}Kit^{+}) bone marrow cells from *in vivo* treated mice (n=6/treatment). Average ±SEM expression of **(D)** *Kit* and **(E)** *Sca1* in c-Kit^{+} bone marrow cells from *in vivo* treated mice (n=6 vehicle, n=5 all remaining treatments). **(F)** Average ±SEM proportion of CD45.2^{+} myeloid cells (CD11b^{+}Gr1^{-}, CD11b^{-}Gr1^{+}, CD11b^{+}, Gr1^{+}) in the bone marrow of treated mice (n=6/treatment). Average ±SEM expression of **(G)** *Bcl2,* **(H)** *Flt3*, and **(I)** *Myc* in c-Kit^{+} bone marrow cells from *in vivo* treated mice (n=6 vehicle, n=5 all remaining treatments). For panels B-I, significance determined by one-way ANOVA with Tukey's multiple comparisons test. Individual data points represent biological replicates. ****p<0.0001, ***p<0.001, **p<0.01, *p<0.05.

Table S1. ***FLT3*****-ITD AML patient sample characteristics.**

Biobank Identifier	Sex	New/Relapsed	Additional Mutations	Cytogenetics
T-20-015	F	New	NPM1, TET2, JAK2	del(15q)(17q)(21q)
T-21-012	M	New	DNMT3A, NPM1, TET2	Normal
T-21-018	F	New	CEBPA, IDH2, NPM1	Normal
T-21-042	M	New	TET2, U2FAF1, WT1	Normal
T-21-068	M	New	NPM1, TET2, KMT2A	Normal
T-22-021	M	New	DNMT3A, IDH1	-17, dup(3q26)x3, del(7q)(17p)
T-22-023	M	New	DNMT3A, SMC3	del(21q)
T-22-028	F	New	NPM1, WT1, DNMT3A, GATA2	Normal
T-24-005	M	Relapsed	FLT3del, NPM1, SRSF2, TET2	Normal
T-25-010	M	New	BCOR, BCORL1, RUNX1, U2AF1, SMC3	Normal

Data S1. (separate file)
in vitro RNA-seq and H3K27ac ChIP-seq analyses in CCS1477 treated *D/F* and *T/F* AML cells.

Figure 2G

Top: Myc

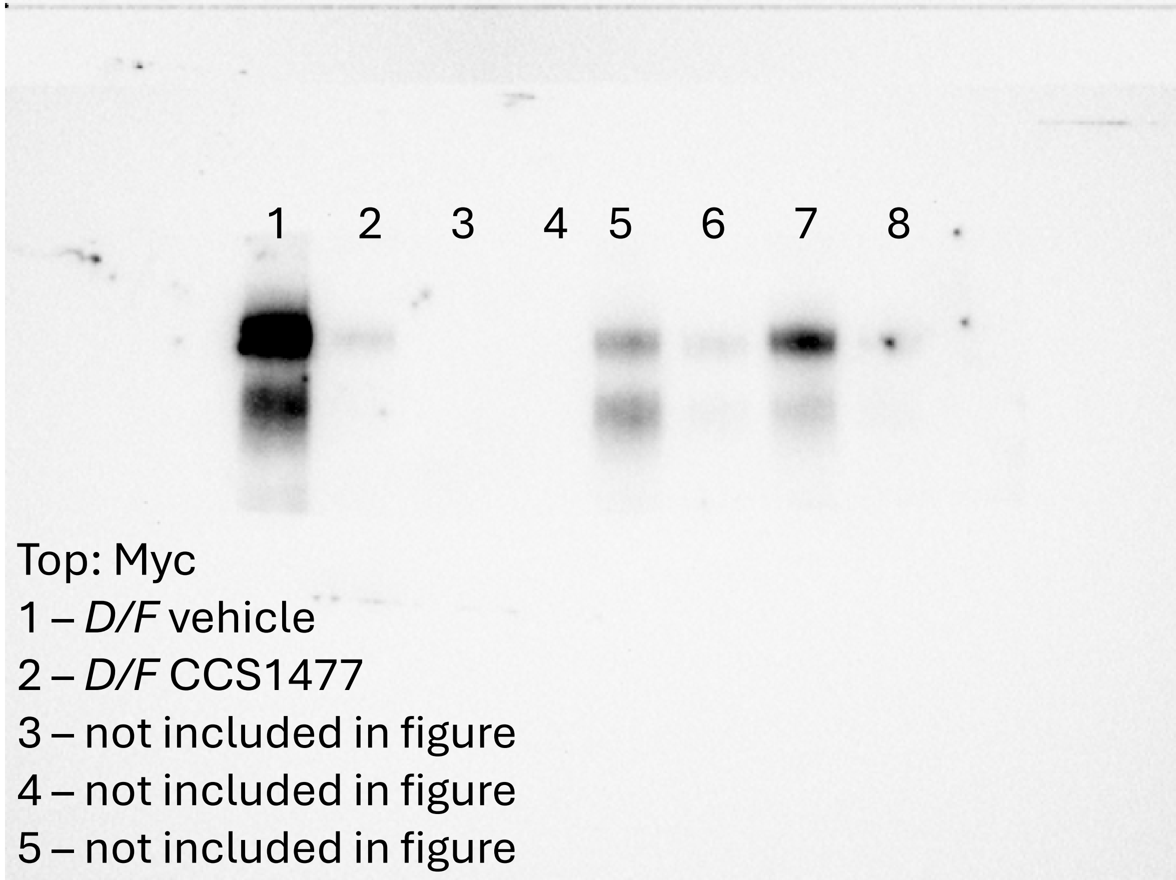

Top: Myc
1 – *D/F* vehicle
2 – *D/F* CCS1477
3 – not included in figure
4 – not included in figure
5 – not included in figure
6 – not included in figure
7 – *T/F* vehicle
8 – *T/F* CCS1477

Bottom: β-actin

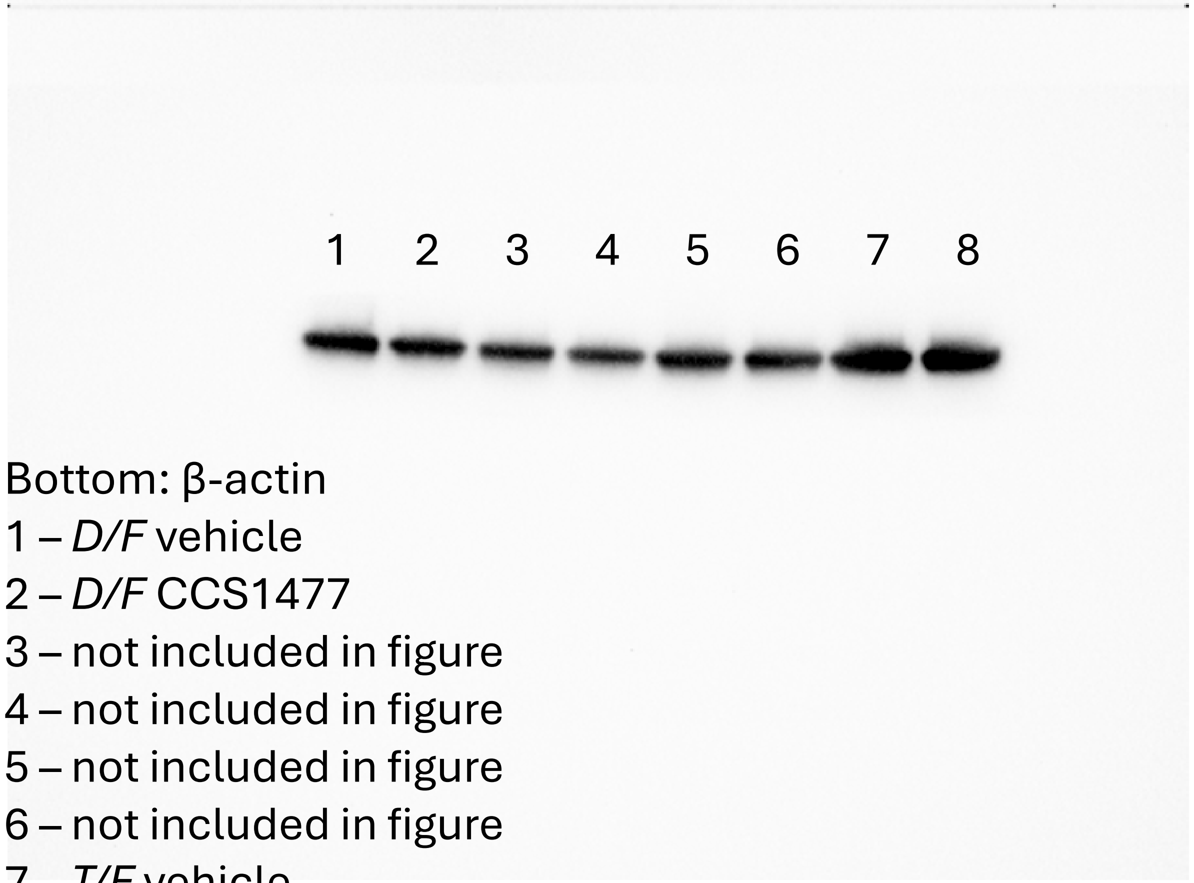

Bottom: β-actin
1 – *D/F* vehicle
2 – *D/F* CCS1477
3 – not included in figure
4 – not included in figure
5 – not included in figure
6 – not included in figure
7 – *T/F* vehicle
8 – *T/F* CCS1477

Lanes 1,2 were duplicated. Lanes 7 and 8 will replace Figure 2G bottom right

Supplementary Figure S2B

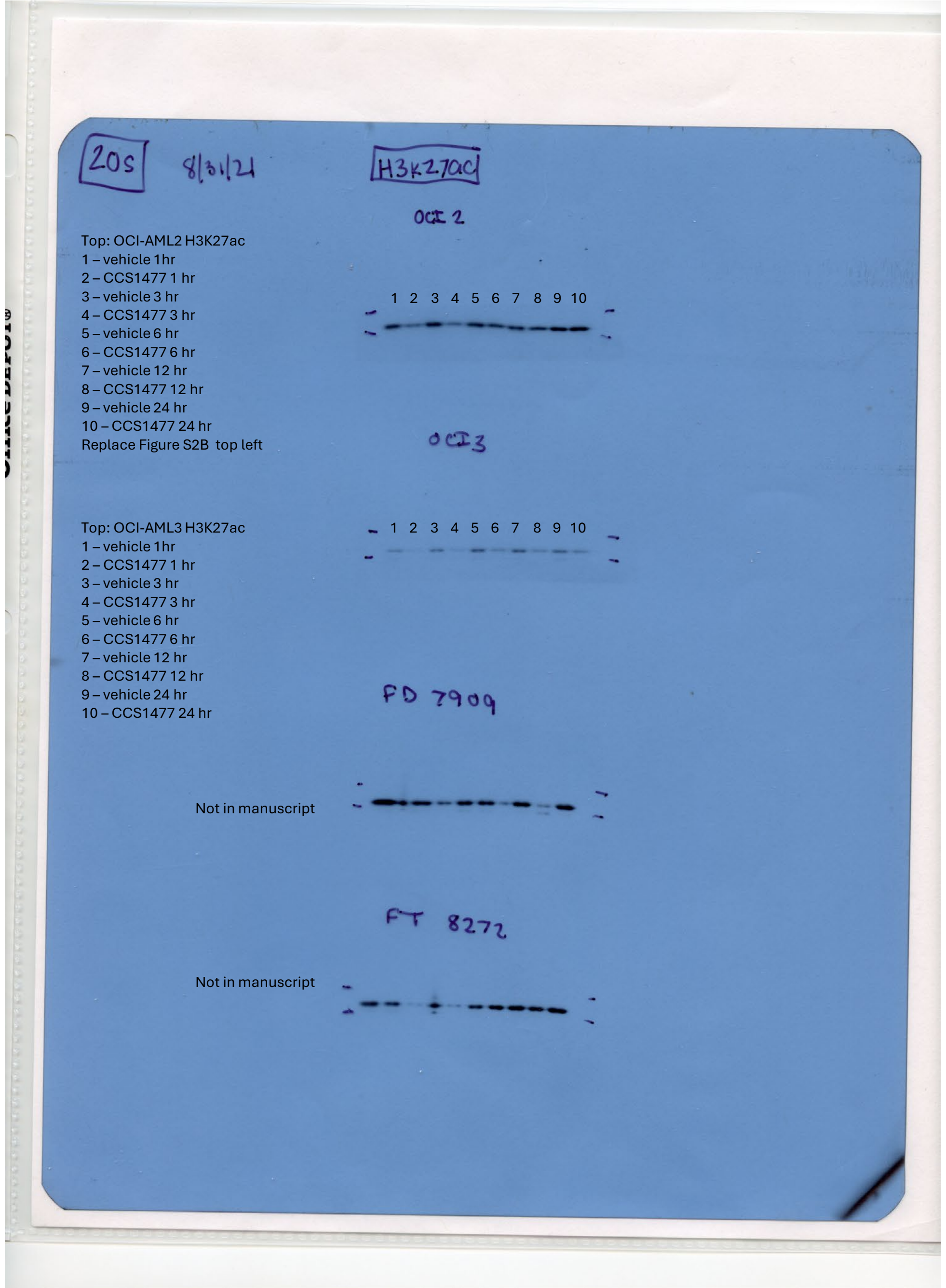

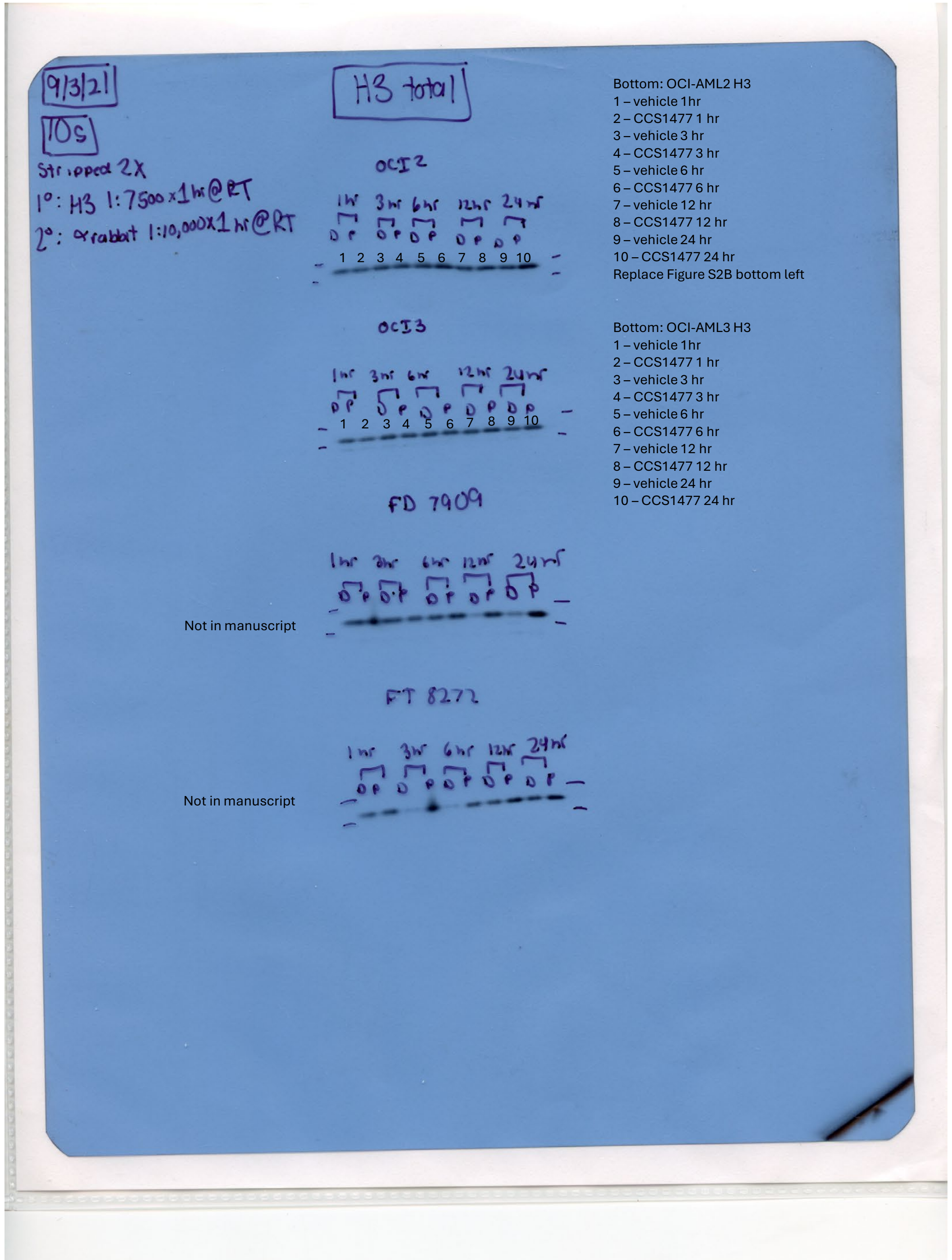

Supplement: Supplementary file 1 — Figs. S1 to S3 Table S1 Legend for data S1 Uncropped Western blots [file sciadv.aec9305_sm.pdf]
